# 600 A Meta-analysis of Expected In-patient Burn Mortality Rates

**DOI:** 10.1093/jbcr/irae036.234

**Published:** 2024-04-17

**Authors:** Margarita S Elloso, Diana Tedesco, Marc G Jeschke

**Affiliations:** Hamilton General Hospital, West Lincoln, ON; Hamilton Health Sciences Corporation, Hamilton, ON; Hamilton Health Sciences, Hamilton, ON; Hamilton General Hospital, West Lincoln, ON; Hamilton Health Sciences Corporation, Hamilton, ON; Hamilton Health Sciences, Hamilton, ON; Hamilton General Hospital, West Lincoln, ON; Hamilton Health Sciences Corporation, Hamilton, ON; Hamilton Health Sciences, Hamilton, ON

## Abstract

**Introduction:**

Improvements in burn care have significantly decreased burn-related mortality over the last several decades. However, a small percentage of patients admitted to burn centers still die from their burn injuries. There is a paucity of information as to the rate of burn deaths at specific time points. The purpose of this study was to determine an updated estimate of overall burn mortality as well as expected event rates within 30-, 60- and 90- days post-burn admission.

**Methods:**

A search of the existing literature was conducted using PubMed and Google Scholar databases in December 2022. An additional search of relevant primary literature and review articles was performed. Title and abstract screening was performed, followed by full-text reviews. Studies related to burns including patients ≥ 16 y/o with sample sizes ≥ 250 reporting overall in-hospital mortality and mortality at 30-, 60- and/or 90- days post-admission were included in the analysis. Studies including pediatric populations in analyses and patients with non-burn etiologies were excluded. A secondary analysis of adult patients admitted to our burn centre between 2006-2021 were included.

**Results:**

A total of selected 9 studies were included in the meta-analysis (4 retrospective, 1 prospective, 1 double-blind, randomized, placebo-controlled trial, and 1 Phase III, multi-center, open label, investigator-initiated, randomized trial) in addition to 2611 patients admitted to our burn centre. A total of 10,824 patients were included in our analysis.

The overall mortality rate of the included studies is 8.51%. By 60-days post-admission, the overall mortality rate of 6 studies was 7.51%. By 90-days post-admission, the overall mortality rate is 11.66%.

The expected mortality rate at 30 days is 6.62%, at 60 days 2.99%, and at 90 days 0.91%. The mortality rate within the first 30 days of admission is 6.6% (95% CI 6.3-6.9%). A decreasing trend in mortality is noted at 60 days of admission which is 1.64% (95% CI 1.4-2%), 90 days of admission 0.5% (95% CI 0.43-0.67%), and 120 days of admission 0.3% (95% CI 0.17-0.37%).

**Conclusions:**

The meta-analysis shows a higher rate of mortality during the first 30 days of burn admission followed by a decreasing trend in mortality at 60- and 90-days post-admission.

**Applicability of Research to Practice:**

Our findings suggest further inquiry into factors contributing to increased mortality within the first 30 days of hospital admission as well as the development of effective therapeutic strategies to decrease the rate of mortality.

